# Changes in Red Blood Cell Properties and Platelet Function during Extracorporeal Membrane Oxygenation

**DOI:** 10.3390/jcm9041168

**Published:** 2020-04-19

**Authors:** Annemieke Oude Lansink-Hartgring, Roland Hoffmann, Walter van den Bergh, Adrianus de Vries

**Affiliations:** 1 Department of Critical Care, University of Groningen, University Medical Center Groningen, 9700 RB Groningen, The Netherlands; w.m.van.den.bergh@umcg.nl; 2 Department of Cardiothoracic Surgery, University Medical Center Groningen, 9700 RB Groningen, The Netherlands; r.f.hoffmann@umcg.nl; 3 Department of Anesthesiology, University Medical Center Groningen, 9700 RB Groningen, The Netherlands; a.j.de.vries@umcg.nl

**Keywords:** extracorporeal membrane oxygenation, bleeding, platelet function

## Abstract

Extracorporeal membrane oxygenation (ECMO) is associated with frequent hemorrhagic and thromboembolic complications. The multiple effects of ECMO include inflammatory response on contact with the circuit; hemolysis acquired von Willebrand syndrome likely affects the function of red blood cells (RBC) and platelets. The aim of this prospective observational study was to analyze RBC aggregation and elongation (deformability) and platelet aggregation in the first week of ECMO. Sixteen patients were included. Blood samples were taken prior to initiation of ECMO and on days 1, 2, 3, 5, and 7. RBC aggregation and elongation were analyzed using the laser-assisted optical rotational red cell analyzer (Lorrca). Upstroke, top, and amplitude as indices of aggregation showed significant time effects. RBC elongation was not affected at low shear stress. At high shear stress there was an increase in the elongation index at day 2 (*p* = 0.004), followed by a decrease. Platelet function was analyzed using multiple electrode aggregometry (Multiplate^®^). In pairwise comparison in the days 1–7 to the value prior to ECMO there was no significant difference in platelet aggregation by any of the three agonists (ADP *p* = 0.61; TRAP *p* = 0.77; Ristocetin *p* = 0.25). This implies that the rheology of RBCs seemed to be more affected by ECMO than platelets. Especially the red blood cell deformability continues to decline at higher shear stress.

## 1. Introduction

Extracorporeal membrane oxygenation (ECMO) is increasingly used in the intensive care unit (ICU) and has improved the survival of patients with critical respiratory and/or circulatory failure. However, complications of ECMO treatment are frequent. Especially the incidence of hemorrhage (15%–20%) and to a lesser extent thromboembolic events (2%–4%) during ECMO treatment are significant [1]. This may in part be caused by the effect of ECMO on the function of red blood cells and platelets. Exposure of blood to the surface of the ECMO circuit initiates the contact factor pathway, activates platelets, and induces an inflammatory response. As a consequence, hemostasis is disrupted, coagulation is promoted, and in the absence of anticoagulation, a thrombus may form, resulting in device failure or embolic stroke. Anemia is also frequent during ECMO treatment due to (postoperative) blood loss or hemolysis. Hemolysis as an expression of membrane instability may be preceded by changes in aggregation and deformability of the red blood cells (RBCs) [2]. Such changes may also affect the ability of RBCs to interact in normal coagulation and thus result in hemorrhage. On the contrary, free hemoglobin was found to promote platelet adhesion and formation of micro thrombi on Von Willebrand factor, indicating that free hemoglobin is associated with thrombotic events [3]. Alterations in RBC rheology (reduced deformability and increased aggregation) were demonstrated in a prospective study in septic patients and reductions in RBC deformability over time are associated with a poor outcome [4]. It is therefore necessary to analyze both platelet function and RBC properties during ECMO. The extreme deformability of RBCs plays a role in the microcirculation. RBCs can pass through capillaries with a diameter of approximately half the diameter of the RBC in rest. This property is also responsible for the surprisingly low viscosity at high shear rates in the large arteries under normal circumstances when RBCs take up almost 50% of the volume of whole blood. It can therefore be expected that even small increases in RBC rigidity might lead to significant disturbances in the microcirculation [5]. Poor microcirculation at initiation of veno-arterial (VA) ECMO and the inability to restore the microcirculation during the first 24 h is associated with mortality [6,7]. Thrombocytopenia in patients on ECMO is frequent but the severity of the underlying illness and platelet count at the time of ECMO cannulation seem to be more important risk factors for bleeding [8]. Acquired von Willebrand syndrome and impaired platelet function is also described during VV ECMO support and may be more associated with hemorrhagic complications than the absolute platelet count [9,10].

Insight into the interaction between red blood cells, platelets and vascular wall may help to prevent these bleeding complications and to decrease thrombotic events. Therefore, it is likely that ECMO therapy affects both RBC and platelet function after its initiation, however, the time course of these changes and their clinical relevance is yet unclear. We hypothesized that RBC rheology and platelet function changes over time after the start of ECMO. The aim of the current study was to analyze RBC function, measured by aggregation and elongation (deformability) and platelet function measured by aggregation in the first week of ECMO.

## 2. Experimental Section

### 2.1. Trial Design and Patients

We conducted a single center prospective observational study at University Medical Center Groningen, the Netherlands, functioning as an ECMO referral center. The study was approved by the Medical Ethical board (METc 2017/550). Written informed consent was obtained.

### 2.2. Study Population

Inclusion criteria were patients aged 18 years or older requiring ECMO. Exclusion criteria were subsequent start of VA ECMO after the use of cardiopulmonary bypass (unless there was a measurement done prior to the procedure as baseline), pregnancy and use of ECMO only for support during high risk procedure like percutaneous cardiac intervention with planned removal of ECMO within 24 h.

### 2.3. ECMO Management

ECMO was provided with a Cardiohelp^®^ system (Maquet Critical Care, Solna, Sweden) equipped with a HLS Set Advanced 7.0 with a biocompatible BIOLINE Coating (Maquet). Unfractionated heparin (heparin Leo^®^, Ballerup, Denmark) was used for anticoagulation with a target activated partial thromboplastin time between 50 and 70 s.

### 2.4. Blood Sampling

A baseline blood sample was obtained from the arterial line prior to ECMO initiation. Additional samples were collected the next day (day 1) and at days 2, 3, 5, and 7, if the patients were still receiving ECMO support. Samples were taken from the arterial line and anticoagulated in 3 mL hirudin tubes (Roche, Basel, Switzerland).

### 2.5. Laser-Assisted Optical Rotational Red Cell Analyzer

**Figure 1:**
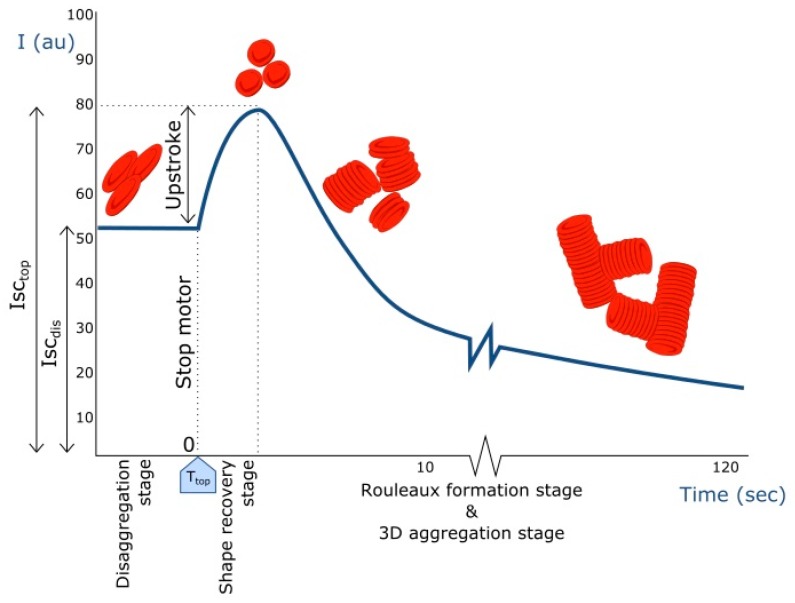
Syllectogram of red blood cell (RBC) aggregation: The intensity of laser backscatter (Isc) plotted versus time before and after a sudden stop of the motor. RBC morphology, corresponding to the various parts of the curve, is indicated (see text).

Both RBC aggregation and deformability as determinants of the blood viscosity were determined by a laser-assisted optical rotational red cell analyzer (Lorrca, RR Mechatronics, Hoorn, The Netherlands) [11]. The blood samples were measured without hematocrit correction. During measurement, each sample was sheared under rotation at 37 °C in a concentric-cylinder system with a gap of 0.3 mm between the cylinders. A syllectogram was created by plotting the laser backscattered intensity, measured by a photodiode, versus time (Figure 1). The reflection was measured over a period of 120 s at a rate of 100 samples per second. First, complete disaggregation of the RBCs was induced by applying a high shear rate. This is reflected in the initial laser backscattered intensity (Figure 1). An abrupt stop of the motor caused the elongated and orientated RBCs to retake their normal, biconcave, shape and orientate randomly. This is reflected in the upstroke, and then the aggregation process starts, at which point RBCs form large stacks (rouleaux) reflected in the amplitude. The amplitude may be combined with the time to obtain a single number: the aggregation index (AI). For the measurement of RBC deformability, the RBCs are elongated under various shear rates that lead to shear stresses from 0 to 49.84 Pa. The elongation index (EI) is used to estimate the ability of RBC deformation by calculating the ratio of long and short axes of the deformed RBCs at the preset shear rates. We present data for a low (0.57 Pa) and a high (7.49 Pa) shear stress.

### 2.6. Multiple Electrode Aggregometry

Platelet function analysis was performed with the Multiplate^®^ Analyzer (Roche Diagnostics International Ltd., Munchen, Germany). This system measures impedance changes as platelets aggregate between two double-sensor electrodes immersed in a cuvette. The increase in impedance is transformed into aggregation units (AU) and plotted against time on the computer screen. Hirudin anticoagulated whole blood rested for a maximum of 30 min before analysis. Whole blood was diluted with isotonic saline 1:1 followed by three minutes of incubation at 37 °C. The platelet aggregation response was studied with adenosine diphosphate (ADP, 6.4 μM), a direct platelet activator, with thrombin receptor activating peptide (TRAP, 32 μM), a strong direct activator to assess the maximal activation response, and with high dose ristocetin (Risto, 50 μL, 0.77 mg/mL), an indirect activator which induces von Willebrand factor mediated platelet activation, which might be more affected by ECMO.

### 2.7. Statistics

Since there were no data available for the effects of ECMO on Lorrca, no formal sample size calculation was performed for this explorative analysis. Categorical variables are reported as frequencies and percentages. Normally distributed data are described as mean ± standard deviation. Not normally distributed data are presented as median with interquartile range (IQR). For comparisons we used the Mann–Whitney test. As we had missing values, for example due to early ECLS withdrawal, we used for the repeated measurement analysis a parsimonious linear mixed model based on Aikake’s information criterion with time as fixed effect and compound symmetry as covariate structure (SPSS version 27). Individual time points were compared to baseline samples. A *p*-value less than 0.05 was considered significant.

## 3. Results

### 3.1. Population

**Figure 2:**
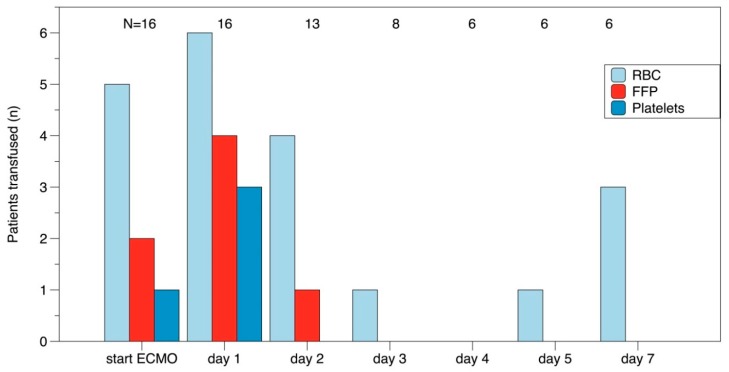
Transfusion of blood products. Horizontal axis shows days on extracorporeal membrane oxygenation (ECMO). Vertical axis shows number of patients transfused. The numbers in the upper part of the graph depict the patients on ECMO on that day.

We studied 16 consecutive patients treated with ECMO in our ICU between July 2018 and August 2019. Patient’s demographics are presented in Table 1. The hemoglobin concentrations fell from 6.7 ± 1.6 mmol/L just before initiation of ECMO to 5.4 ± 1.0 mmol/L on the first day and stabilized thereafter on 4.4 ± 0.5 mmol/L. Platelet count just prior to initiation of ECMO was 223 ± 101 × 10^9^/L which decreased to 187 ± 103 on day 1. D-dimers increased from 6795 ± 12,878 μg/L at day 1 of ECMO to 22,129 ± 25,901 μg/L at day 7. The number of patients who received transfusion of blood products is shown in Figure 2.

### 3.2. Laser-Assisted Optical Rotational Red Cell Analyzer

#### 3.2.1. Red Blood Cell Aggregation

**Figure 3:**
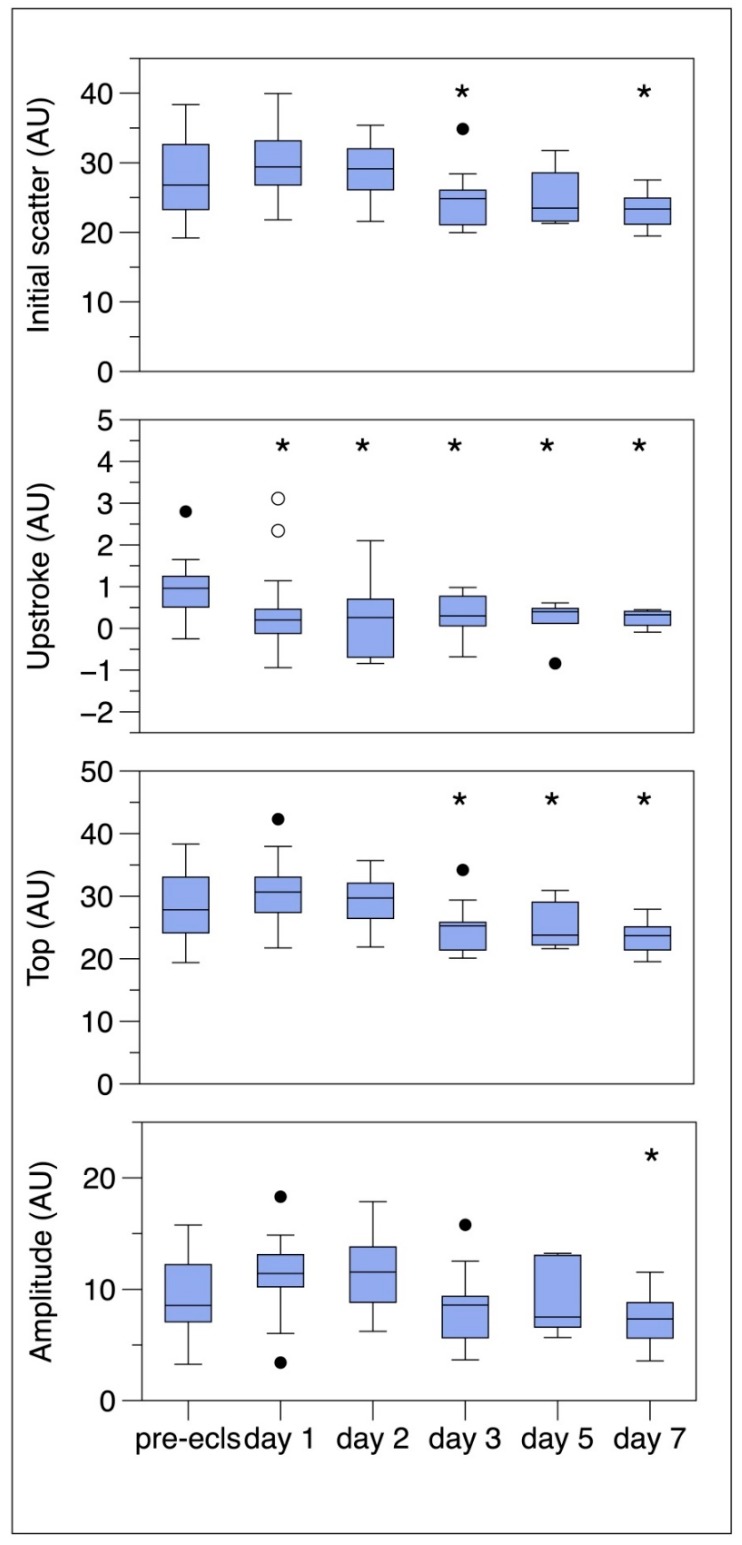
Changes over time in the components that form the syllectogram with the laser-assisted optical rotational red cell analyzer. The initial scatter reflects the complete disaggregation of the RBCs at a preset shear stress. The upstroke reflects that the RBCs resume their normal biconcave shape. This culminates in the top from which the aggregation process starts. The complete aggregation is reflected in the amplitude. Boxes represent the interquartile range (25%–75%), dots represent the outliers. * *p* = 0.05 indicating significant difference with pre ECLS value. AU = Arbitrary Units. ECLS = extracorporeal life support.

There were significant time effects for the initial scatter intensity (*p* = 0.003), the top of the laser backscatter intensity (*p* = 0.001), the upstroke (*p* = 0.001), and the amplitude which is the total extent of the aggregation (*p* = 0.015) in Figure 3. All these parameters are part of RBC aggregation, which combined with the time may be calculated and result in the AI. This calculated index showed a trend towards decrease in the first week on ECMO, but this time-effect was not significant (*p* = 0.110).

#### 3.2.2. Red Blood Cell Deformability

**Figure 4:**
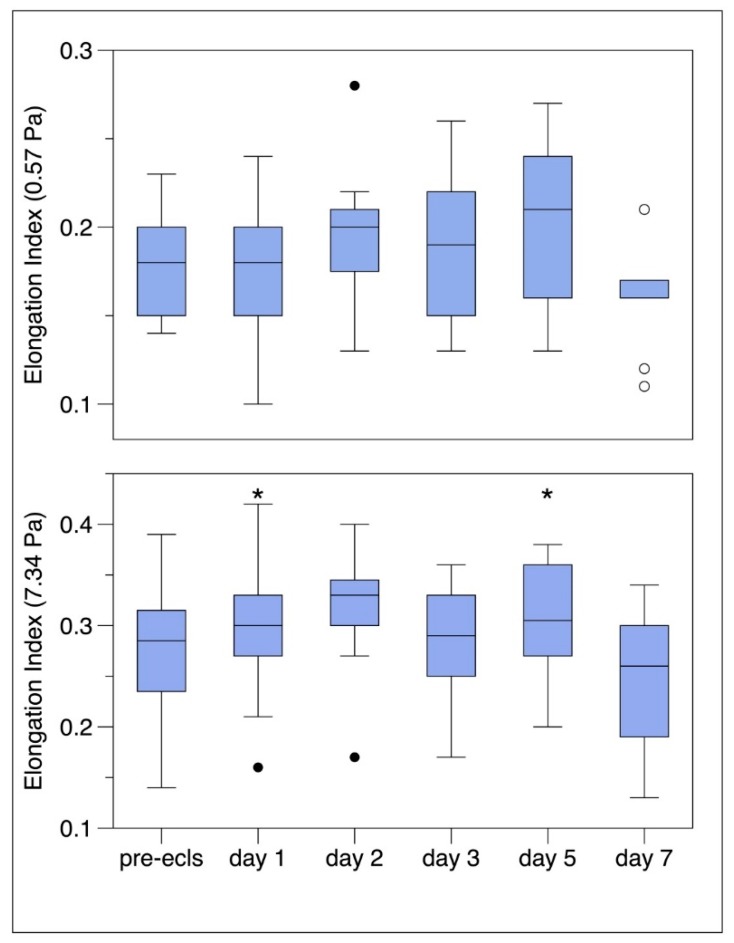
Elongation index at low shear stress (0.57Pa) and elongation index at high shear stress (7.34 Pa). Boxes represent the interquartile range (25%–75%), dots represent the outliers. * *p* = 0.05 indicating significant difference with pre ECLS value. ECLS = extracorporeal life support.

After a small increase with a maximum on day 2 there was a slight decrease in EI. At all time points the measurements showed alteration in RBC deformability (Figure 4). The EI reached a maximum of 0.3 and decreased at a shear stress of 7.34 Pa. In a pairwise comparison to the value prior to ECMO there was no significant difference in the values of the elongation index in the days 1 through 7 at low shear stress. Although there was a significant time effect (*p* = 0.041) in univariate testing. In a pairwise comparison to the value prior to ECMO there was a significant difference in the value of the elongation index at day 2 (*p* = 0.004) at high shear stress. There was also a significant time effect (*p* = 0.004) in univariate testing.

### 3.3. Platelet Function Analysis with Multiplate

**Figure 5:**
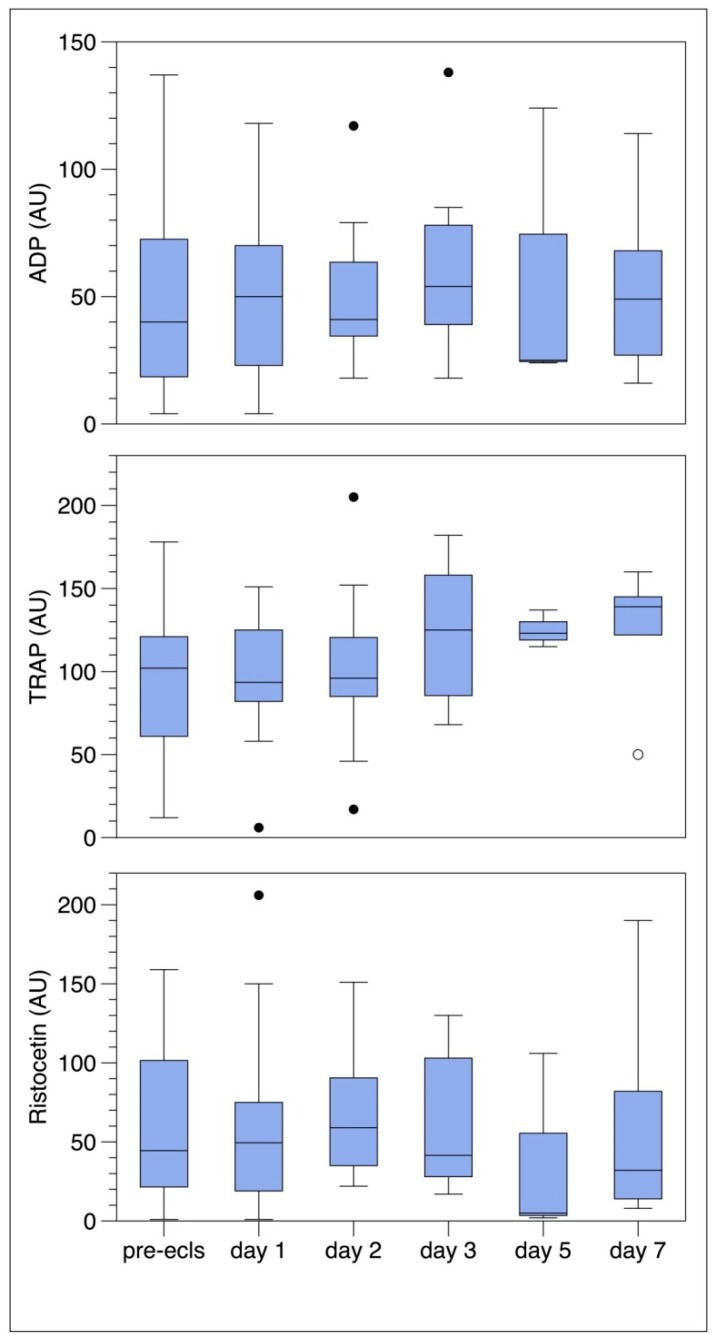
Platelet aggregation analysis with Multiplate after stimulation with ADP, TRAP and Ristocetin prior to start of ECMO and on days 1, 2, 3, 5, and 7. Boxes represent the interquartile range (25%–75%), dots represent the outliers. ECLS = extracorporeal life support.

In Figure 5 the results of the platelet function analysis are presented. The median value of ADP prior to ECMO was 43 AU (IQR 19–71), below the reference range (53–122 AU). For TRAP the median value prior to ECMO was 99 AU (IQR 60–129), within the reference range (94–156 AU). For the indirect activator Ristocetin, the median value prior to ECMO was far below the reference range of 90–201 AU, with 34 AU (IQR 20–99). In pairwise comparison in the days 1–7 to the value prior to ECMO there was no significant difference in platelet aggregation by any of the three agonists (ADP *p* = 0.61; TRAP *p* = 0.77; Ristocetin *p* = 0.25).

## 4. Discussion

The most important finding of our study is that the rheology of red blood cells seems to be more affected by ECMO than platelet function. Especially the red blood cell deformability continues to decline at higher shear stress, however, the effect seems minor.

To gain more insight into the interaction of ECMO and RBCs, we focused on the components that form the syllectogram, more than the composed AI. The AI did not significantly alter over time, but all components that make up the AI did. The basic laser scatter intensity, the upstroke, and the amplitude all significantly decreased over the time points. The decrease in upstroke points towards less disaggregation into single RBCs while the amplitude which is the total extent of the aggregation points to less forming of rouleaux during ECMO. Both findings suggest that the membrane properties of the RBCs are affected by ECMO. However, and this is a limitation of our study, hemoglobin levels also decreased during ECMO treatment and there may be a causal relationship with the lowering of the basic laser scatter intensity, upstroke and hematocrit levels. Hardeman reported that the total extent of RBC aggregation as measured with the Lorrca, was found to be nonlinear dependent on hematocrit with an optimum between 0.42 and 0.46 L/L, in line with the findings of Deng et al., obtained with light transmission technology [11]. The AI increased steadily over the hematocrit range 0.18 to 0.78 L/L [12]. We consider this a less likely explanation for our findings as our hemoglobin levels remained in a reasonably narrow and clinically acceptable range.

One of the stronger points of our study is that aggregation and deformation of RBCs in ECMO patients has not been studied before. In patients undergoing cardiac surgery with the use of cardiopulmonary bypass the use of mechanical cell saver did not alter the AI but led to a significantly lower EI, meaning that the mechanical cell saver seemed to add additional shear stress and led to decreased deformability [13]. Combined with our results this suggests that mechanical treatment of blood affects the membrane properties, but our study does not allow us to assess the degree of microcirculatory impairment.

Using an ektacytometer, it was shown that cardiac surgery with cardiopulmonary bypass is associated with a dose-dependent decrease in RBC deformability when stored allogeneic RBCs were transfused; this effect was not seen with autologous salvaged RBCs [14]. A number of our patients also received allogeneic transfusions and this may have affected our results. However, it would be unethical to withhold the blood transfusions given our restrictive transfusion policy. Given our small cohort we did not perform a separate analysis of transfused and nontransfused patients.

In the sepsis study using the Lorrca, the AI in septic patients was higher than in healthy volunteers and nonseptic ICU patients. RBC deformability was already reduced in septic patients at ICU admission and worsened in nonsurvivors. In nonseptic patients these changes were not observed [4]. This effect in aggregation might be related to higher fibrinogen levels in sepsis patients as fibrinogen is needed to form bridges between the RBCs and facilitate aggregation but this was not shown. In our patients the AI showed a trend towards lowering on ECMO while the fibrinogen levels remained stable. The deformability is reduced as shown in the relatively low maximum of the elongation index and the significant time effect at high(er) shear stress. This might have a detrimental impact on the microcirculation. We did not find a time-effect for platelet function during ECMO with the multiplate suggesting a functional balance in the light of developing (mild) thrombocytopenia. This effect was also seen in a study in twenty patients on VV ECMO where platelet function was impaired prior to the ECMO initiation; ECMO led to a further impairment but after two days the values recovered to baseline [15]. In a study with both VV and VA ECMO patients it was shown that the platelet aggregation was not impaired when the results were interpreted considering the low platelet counts [16].

Studies on ECMO patients are limited in interpretation due to the heterogeneous population with a variety of underlying illness leading to the need for ECMO support. The results of the present study are confounded by blood loss and transfusions of RBCs, plasma, and platelets. Multiplate might not be the most appropriate analyzer for platelet dysfunction on ECMO. Most studies, including ours, are influenced by developing thrombocytopenia during ECMO. The extent of the influence of hemoglobin oxygenation grade on the results of the Lorrca is not exactly known but probably negligible [5].

## 5. Conclusions

Although ECMO caused only minor changes in RBC properties, there was a trend towards a reduced deformability and aggregation of the red blood cells over time during extracorporeal membrane oxygenation. This time dependent effect was not seen in platelets.

## Figures and Tables

**Table 1:** Patient characteristics at baseline.

Patients (*n*)	16
Age, mean	50 ± 12
Gender, male	10 (63%)
Body mass index (kg/m^2^), mean	26 ± 4
ECMO modus, VA (%)	9 (56%)
Diagnosis:	
Pulmonary	7 (44%)
Cardiac	6 (37%)
ECPR	3 (19%)
APACHE IV score, mean	101 ± 35
Duration of ECMO, days median (IQR)	5.5 (2.25–14.5)
Successful weaning of ECMO	7 (44%)
Hemoglobin, prior to start ECMO, mean	6.7 ± 1.6 mmol/L
Platelets, prior to start ECMO, mean	223 ± 101 × 10^9^/L
D-dimers, on the first day of ECMO, mean	6795 ± 12,878 μg/L
Fibrinogen, on the first day of ECMO, mean	4.4 ± 2.7 g/L

ECMO: extracorporeal membrane oxygenation; VA: veno-arterial; ECPR: extracorporeal life support; APACHE: acute physiology and chronic health evaluation.
